# The Role of Loss‐of‐Function *KCNH2* Variants in Cardiac Arrhythmias, Seizures and the Risk of Sudden Unexpected Death in Epilepsy

**DOI:** 10.1111/jnc.70441

**Published:** 2026-04-14

**Authors:** Hian M. Lee, Xue N. Gan, Khaing Phyu Aung, Ian C. Forster, Christopher A. Reid, Ming S. Soh

**Affiliations:** ^1^ The Florey Institute of Neuroscience and Mental Health University of Melbourne Parkville Victoria Australia; ^2^ Faculty of Medicine and Health Sciences University Malaysia Sabah Kota Kinabalu Malaysia

**Keywords:** biomarkers, cardiac arrhythmia, epilepsy, genetics, ion channels, KCNH2, SUDEP

## Abstract

Sudden Unexpected Death in Epilepsy (SUDEP) is the leading cause of mortality in patients in epilepsy, yet its underlying mechanisms are poorly understood. Emerging evidence suggests a significant role for genetic factors that influence cardiac function in SUDEP risk, particularly loss‐of‐function variants in *KCNH2*, which encodes the K_v_11.1 potassium channel. K_v_11.1 channels are expressed in both cardiac and neuronal tissues. Pathogenic *KCNH2* variants are strongly associated with cardiac arrhythmias leading to increased risk of sudden cardiac death. There is also evidence that *KCNH2* variants can influence seizure susceptibility. Furthermore, K_v_11.1 is expressed in brain autonomic and cardiorespiratory centres, where its impairment may compromise autonomic function, including breathing. Therefore, changes in K_v_11.1 channel function in both central and cardiac tissues could potentially contribute to increased SUDEP risk. In this review, we explore the potential dual contribution of K_v_11.1 channel dysfunction to SUDEP risk. We hypothesise how this dual‐system vulnerability may predispose individuals with pathogenic *KCNH2* variants to both cardiac arrest and respiratory failure following seizures. By integrating genetic, electrophysiological, and neuroanatomical evidence to support our hypothesis, this review presents a multidisciplinary framework for understanding SUDEP and highlights the potential of pathogenic *KCNH2* variants as a biomarker for risk and targeted intervention.

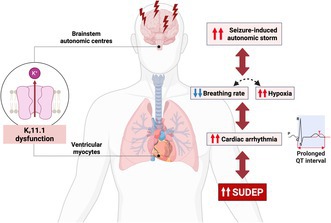

## Graphical abstract

Seizures can trigger a surge in brain‐driven autonomic signals, causing dangerous changes in heart rhythm and breathing. This ‘storm’ increases stress on the heart, leading to prolonged QT intervals and arrhythmias, while also slowing breathing and causing low oxygen levels. When these effects occur together, they create a vicious cycle that can result in sudden unexpected death in epilepsy (SUDEP). This review highlights how genetic changes in the K_v_11.1 potassium channels could potentially exacerbate this risk.

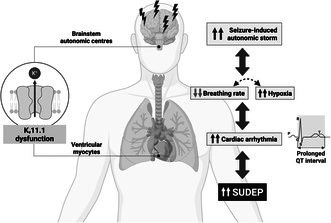

**Abbreviations**
ECG: electrocardiogram
EEG: electroencephalogram
GABA: γ‐aminobutyric acid
GTCS: generalised tonic–clonic seizures
*HERG*
: *human‐ether a‐go‐go‐related gene*
I_Kr_
: delayed rectifier K_v_11.1‐mediated current
LQT2: long‐QT syndrome type 2
LQTS: long‐QT syndrome
MORTEMUS: MORTality in Epilepsy Monitoring Unit Study
PAS: Per‐Arnt‐Sim
SUDEP: sudden unexpected death in epilepsy
TdP: Torsades de Pointes
VUS: variants of unknown significance

## 1 Introduction

Patients diagnosed with epilepsy experience recurrent seizures and have a two to threefold increased risk of premature mortality compared to the general population (Thurman et al. 2017). In these patients, sudden unexpected death in epilepsy (SUDEP) is the leading cause of epilepsy‐related premature mortality and second only to stroke in years of potential life lost among the neurological diseases, making it a major public health and economic burden (Thurman et al. 2014). SUDEP is defined as ‘sudden, unexpected, witnessed or unwitnessed, non‐traumatic and non‐drowning death in patients with epilepsy, with or without evidence for a seizure and excluding documented status epilepticus, in which post‐mortem examination does not reveal a toxicologic or anatomic cause of death’ (Nashef et al. 2012). The difficulty of diagnosing SUDEP has led to the following recommended sub‐classifications: Definite SUDEP, definite SUDEP plus (in the presence of a concomitant condition other than epilepsy), probable SUDEP/SUDEP plus (in the absence of autopsy), possible SUDEP (presence of another competing cause), near‐SUDEP/SUDEP plus (patient survives resuscitation after a cardiorespiratory arrest), not SUDEP, and unclassified (Nashef et al. 2012). The underlying mechanism of SUDEP remains elusive but is likely a complex interplay between different factors.

## 2 SUDEP Epidemiology and Risk Factors

The incidence of SUDEP is estimated at about 1.16 cases per 1000 patients with epilepsy (Thurman et al. 2014; Devinsky et al. 2016). However, the number of cases can differ depending on the epilepsy population of study. Epidemiological data indicate a higher incidence among males compared with females (Hesdorffer et al. 2011). SUDEP also appears more prevalent in patients aged between 21 and 50 years old (Devinsky et al. 2016; Thurman et al. 2014). Earlier studies suggested that SUDEP was less frequent in children with epilepsy, with reported rates of approximately 1 in 5000 paediatric patients compared to 1.2 per 1000 adult patients (Harden et al. 2017). However, a more recent population‐based data indicate that SUDEP risk is similar in children and adults, highlighting SUDEP as a lifespan‐relevant complication of epilepsy rather than a predominantly adult phenomenon (Keller et al. 2018). Importantly, children with severe developmental and epileptic encephalopathies, such as Dravet syndrome, exhibit a substantially higher SUDEP risk, with reported incidences of approximately 9 in 1000 patients (Cooper et al. 2016). Uncontrolled generalised tonic–clonic seizures (GTCS) are the most well‐established risk factor for SUDEP. Patients experiencing 1–2 GTCS per year have a 5‐ to 22‐fold increased risk, whereas those with 3 or more GTCS annually face a 15‐ to 32‐fold increase, compared to individuals who have never had GTCS (Sveinsson et al. 2017; Sveinsson et al. 2020). Unsurprisingly, patients with poor adherence to antiseizure medications and pharmaco‐resistant epilepsy are particularly vulnerable to SUDEP (Devinsky et al. 2016; Harden et al. 2017). SUDEP also occurs more commonly at night during sleep, putting patients who live alone at a significantly higher risk (Devinsky et al. 2016; Sveinsson et al. 2017). Presence of comorbidities, especially psychiatric conditions, substance abuse and alcohol dependence, could also increase SUDEP risk by 2‐ to 5‐fold (Sveinsson et al. 2017; Sveinsson et al. 2020).

## 3 Cardiorespiratory Mechanisms Underlying SUDEP

Dissecting the pathophysiological mechanism(s) underlying SUDEP remains a challenge due to the unpredictability of the event. The MORTality in Epilepsy Monitoring Unit Study (MORTEMUS), a systematic retrospective study of SUDEP cases from 147 epilepsy monitoring units worldwide, offered some insights (Ryvlin et al. 2013). 16 SUDEP cases were reviewed, including nine patients with severe epilepsies who were undergoing video‐electroencephalogram (EEG) and electrocardiogram (ECG) monitoring when their terminal generalised tonic–clonic seizures occurred. All nine patients experienced seizure‐induced cardiorespiratory dysfunction, which progressed to respiratory arrest followed by cardiac death (Ryvlin et al. 2013). These findings strongly implicate seizure‐related cardiorespiratory arrest as a central mechanism in SUDEP. Additional clinical and preclinical studies also support this hypothesis, demonstrating that seizures can trigger autonomic dysfunction within the central nervous system, leading to significant disturbances in both cardiac and respiratory function (Aiba and Noebels 2015; Bateman et al. 2010; Dlouhy et al. 2015; van der Linde et al. 2021). This includes changes in cardiac electrophysiology and respiratory depression, which is likely mediated by impaired brainstem regulation.

Furthermore, epilepsy is associated with a 3‐fold increase in the risk of sudden cardiac death (Bardai et al. 2012). Seizures can acutely disrupt cardiac electrophysiology by altering heart rate, inducing arrhythmias and modifying the QT interval, through the impaired regulation the autonomic nervous system (Nei 2009; Ravindran et al. 2016; Baumgartner et al. 2001). Beyond these transient effects, seizures can also cause long‐term changes in cardiac ion channel expression, leading to persistent electrophysiological abnormalities (Li et al. 2019). Changes in cardiac function, especially arrhythmias and QT interval prolongation, are well‐established risk factors for sudden cardiac death (Franciosi et al. 2017). Clinically significant prolongation of the QT interval is a typical characteristic of an inherited arrhythmia condition known as long‐QT syndrome (LQTS), caused by pathogenic variants that alter cardiac ion channel function (Schwartz et al. 2012). The majority of pathogenic LQTS variants are found in three key genes: *KCNQ1* (LQTS type 1), *KCNH2* (LQTS type 2) and *SCN5A* (LQTS type 3). Out of the three, *KCNH2* variants, associated with LQTS type 2 (LQT2) have been most frequently identified in SUDEP cases, presenting in at least 5% of patients, and have been associated with 3‐ to 11‐fold increase in sudden death risk (Table 1) (Bagnall et al. 2016; Bagnall et al. 2017; Tu et al. 2011; Partemi et al. 2013; Soh et al. 2021).

**TABLE 1:** Quantitative summary of human genetic evidence for *KCNH2* in SUDEP.

Study	Cohort design	Sequencing/Analytic scope	*KCNH2* variants identified (counts)	Enrichment/Statistics	SUDEP‐specific vs classical LQT2 evidence
Soh et al. (2021)	Case–control (90 SUDEP vs. 332 non‐SUDEP epilepsy patients)	Gene‐focused extraction of *KCNH2* variants with MAF < 5%, followed by in vitro functional testing to classify LOF vs. no‐change.	SUDEP: LOF 8.9%; no‐functional‐change 2.2%. Controls: LOF 3.3%; no‐functional‐change 2.7%.	LOF variants ~3× enriched in SUDEP vs. epilepsy controls (nominal *p* = 0.04); rare (< 1%) LOF ~11× enriched (nominal *p* = 0.03). LOF R1047L detected in 5.6% of SUDEP vs. 3% of epilepsy controls (gnomAD ~1.8%).	Direct SUDEP signal based on functional LOF burden; distinguishes SUDEP risk enrichment from broader LQT2/SCD literature by using epilepsy controls and functional assays.
Bagnall et al. (2016)	SUDEP exome study with external control exomes. (61 SUDEP vs. 2936 control exomes).	WES with rare‐variant collapsing; targeted screen of cardiac arrhythmia, respiratory control, and epilepsy genes. Population‐based genetic background for burden testing	Aggregated LQTS gene mutations ~7% of SUDEP. *KCNH2* among top 30 genes genome‐wide (*p* = 0.0037). Per‐gene P/LP vs. VUS counts for *KCNH2* not reported.	No gene reached exome‐wide significance; gene‐level signal for *KCNH2* suggests contribution to SUDEP risk (*p* = 0.0037).	Supports SUDEP‐specific genetic contribution in arrhythmia genes including *KCNH2* but does not rely on classical LQT2 SCD cohorts. Complementary to Soh et al. 2021 by using population control exomes rather than epilepsy controls.
Tu et al. (2011)	Post‐mortem SUDEP (forensic centre review 1993–2009). 48 out of 68 SUDEP sequenced, ≥ 340 control alleles genotyped for identified variants in SUDEP cohort.	Sanger sequencing of LQT genes, including *KCNH2*, in the 48 cases. Genotyping of nonsynonymous SUDEP variants in ≥ 340 control alleles.	1/48 sequenced SUDEP carried a previously reported *KCNH2* missense (R176W), which was absent in control alleles (gnomAD 0.03%).	Qualitative rarity only (absence in ≥ 340 control alleles). No population‐frequency analysis performed.	Early molecular‐autopsy evidence. Demonstrates SUDEP‐specific rarity of a *KCNH2* variant.

Abbreviations: LOF = loss‐of‐function, MAF = minor allele frequency, P/LP = pathogenic/likely pathogenic, SCD = sudden cardiac death, VUS = variant of uncertain significance.

## 4 Molecular and Functional Overview of *KCNH2*

**FIGURE 1:**
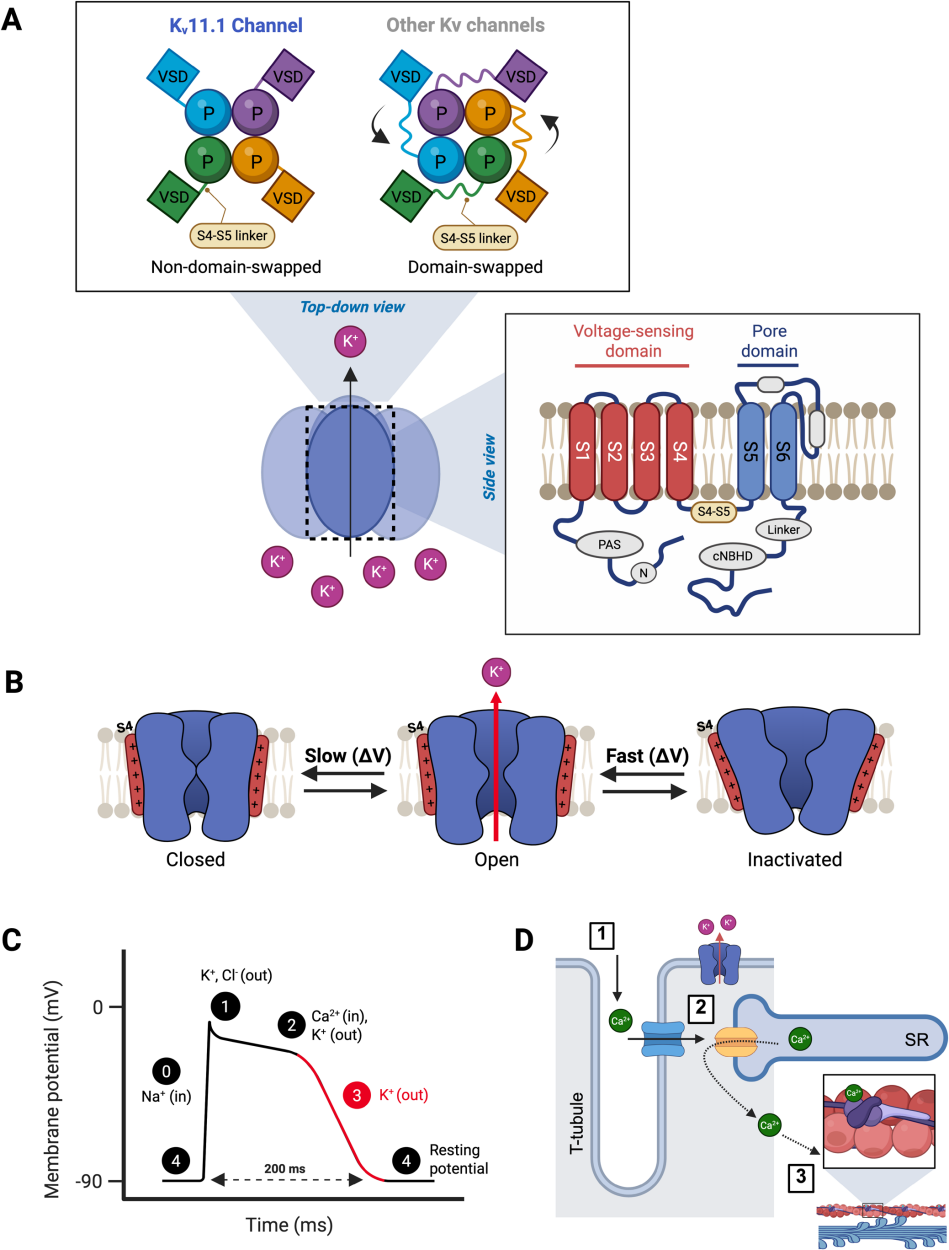
Structure and function of K_v_11.1 channels in cardiac repolarisation. (A) K_v_11.1 architecture compared to other K_v_ channels, showing non‐domain‐swapped arrangement versus domain‐swapped arrangements. Top‐down view shows the tetrameric assembly, while side view illustrates the transmembrane topology with voltage‐sensing (S1‐S4) and pore (S5‐S6) domains. (B) Gating transitions from deactivated (closed) to activated (open) and then to inactivated states with respective voltage dependent kinetics (slow/fast). (C) Membrane potential changes during a cardiac action potential, indicating phases of Na^+^ influx (depolarisation), Ca^2+^ and K^+^ currents (plateau), and K^+^ efflux during phase 3 repolarisation, contributed by K_v_11.1 (red‐highlighted). (D) Integration of K_v_11.1 channel activity within cardiac excitation‐contraction coupling, showing its role in influencing the action potential and maintaining rhythmic contraction. [1] During depolarisation, L‐type Ca^2+^ channels in the T‐tubule open, allowing Ca^2+^ influx. [2] This Ca^2+^ entry activates ryanodine receptors on the SR, triggering a larger Ca^2+^ release into the cytosol. [3] Elevated cytosolic Ca^2+^ binds to troponin C, enabling actin‐myosin cross‐bridge formation and contraction. Loss‐of‐function K_v_11.1 delays repolarisation, prolongs action potential duration and disrupts Ca^2+^ signalling (Spencer et al. 2014), increasing susceptibility to life‐threatening arrhythmias.

*KCNH2*, also known as the *human‐ether a‐go‐go‐related gene* or *HERG*, encodes for the α‐subunit of the voltage‐gated ion channel, K_v_11.1, which is most highly expressed in cardiac tissue but also found in various regions of the brain (Smith et al. 2016; Uhlén et al. 2015; Sjöstedt et al. 2020). Structurally, K_v_11.1 forms a tetramer, with each subunit containing a voltage‐sensing domain (S1–S4) and a pore‐forming region (S5–S6) that allows potassium ion flow (Figure 1A) (Sanchez‐Conde et al. 2022; Warmke and Ganetzky 1994; Sanguinetti and Tristani‐Firouzi 2006; Gustina and Trudeau 2011). The channel has two cytosolic domains, the Per‐Arnt‐Sim (PAS) and cyclic‐nucleotide‐binding homology domains, which interact to modulate channel gating (Cabral et al. 1998; Sanguinetti and Tristani‐Firouzi 2006; Gustina and Trudeau 2011). Notably, structural studies reveal that, unlike other potassium channels, K_v_11.1's voltage sensor is positioned adjacent to its own pore domain rather than being domain‐swapped due to its short S4‐S5 linker (Figure 1A) (Whicher and MacKinnon 2016; Wang and MacKinnon 2017). In the resting state, this short S4‐S5 linker interacts with the C‐terminal of the S6 helix, stabilising the channel in a closed conformation (Ng et al. 2012). This interaction acts as a voltage‐dependent ‘lock’ resembling ligand‐receptor binding and preventing channel opening until depolarisation disrupts the link (Malak et al. 2017; Malak et al. 2019). Upon membrane depolarisation, the S4‐S5 linker disengages from S6 and forms new conformations that favour pore opening. This stability delays the reformation of the original S4‐S5/S6 ‘lock’ interaction, thereby contributing to slow deactivation kinetics (Ng et al. 2012). Meanwhile, despite the channel being structurally open, the narrow selectivity filter region near the extracellular side of the pore undergoes a rapid conformational change that constricts ion flow, leading to fast inactivation (Li et al. 2021). During repolarisation, the voltage‐sensing domains reset, allowing the S4–S5 linker to slowly re‐establish its interaction with S6 and restore the closed conformation (Ng et al. 2012). Together, these gating features underlie K_v_11.1's unique kinetics in cardiomyocytes, where rapid inactivation during depolarisation and slow deactivation upon repolarisation are essential for conducting the rapid component of the delayed rectifier current, I_Kr_, which drives phase 3 ventricular repolarisation (Figure 1B,C) (Sanchez‐Conde et al. 2022; Smith et al. 2016; Vandenberg et al. 2012; Sanguinetti and Tristani‐Firouzi 2006). Phase 3 repolarisation is necessary to allow sufficient time for calcium influx into cardiomyocytes, therefore optimising excitation‐contraction coupling, a process where electrical signals trigger muscle contraction (Figure 1D) (Sanguinetti and Tristani‐Firouzi 2006). Delayed repolarisation due to reduced I_Kr_ prolongs the action potential and Ca^2+^ transients, resulting in abnormal Ca^2+^ signalling (Spencer et al. 2014). In contrast, the delayed rectifier current during phase 3 repolarisation renders the cardiomyocytes temporarily unresponsive to premature stimuli, which helps to reduce the risk of re‐entrant arrhythmias (Sanguinetti and Tristani‐Firouzi 2006).

Beyond its established cardiac role, K_v_11.1 is expressed in neuronal populations throughout the brain, where its distinctive gating kinetics is thought to regulate neuronal excitability and firing patterns (Sjöstedt et al. 2020; Sanchez‐Conde et al. 2022). Its dysfunction has been linked to epilepsy, cognitive deficits and neuropsychiatric disorders (Sanchez‐Conde et al. 2022; Zamorano‐León et al. 2012; Zhou et al. 2023; Huffaker et al. 2009). While the role of pathogenic *KCNH2* variants in arrhythmogenesis and sudden cardiac death is well established, its contribution in epilepsy and SUDEP is less clear. This review explores emerging hypotheses on how K_v_11.1 dysfunction in both cardiac and neuronal tissues may converge to increase SUDEP risk.

## 5 The Role of Loss‐Of‐Function *KCNH2* Variants in Arrhythmias and SUDEP

Loss‐of‐function *KCNH2* variants have been increasingly associated with SUDEP (Table 1), positioning K_v_11.1 as a potential mechanistic link between epilepsy and fatal arrhythmias (Bleakley et al. 2020; Soh et al. 2021; Soh et al. 2025). Loss‐of‐function variants in *KCNH2* are well‐established contributors to LQT2, accounting for 30%–45% of all LQTS cases (Sanchez‐Conde et al. 2022; Smith et al. 2016; Schwartz et al. 2012). These variants impair the rapid delayed rectifier I_Kr_ current, which is essential for phase 3 ventricular repolarisation (Figure 1C). In iPSC‐derived cardiomyocytes modelling LQT2, reduced I_Kr_ has been shown to prolong repolarisation and action potential durations, leading to extended calcium transients and early afterdepolarisation events, which are substrates for life‐threatening arrhythmias (Spencer et al. 2014). Voltage‐calcium dissociation has also been reported in LQTS, in which calcium transients fail to follow membrane voltage changes (Himel et al. 2021). This decoupling destabilises wave front propagation and contributes to Torsades de Pointes (TdP), a dangerous arrhythmia. These findings demonstrate that impaired I_Kr_‐mediated repolarisation due to loss of *KCNH2* function can disrupt excitation‐contraction coupling (Figure 1D).

**FIGURE 2:**
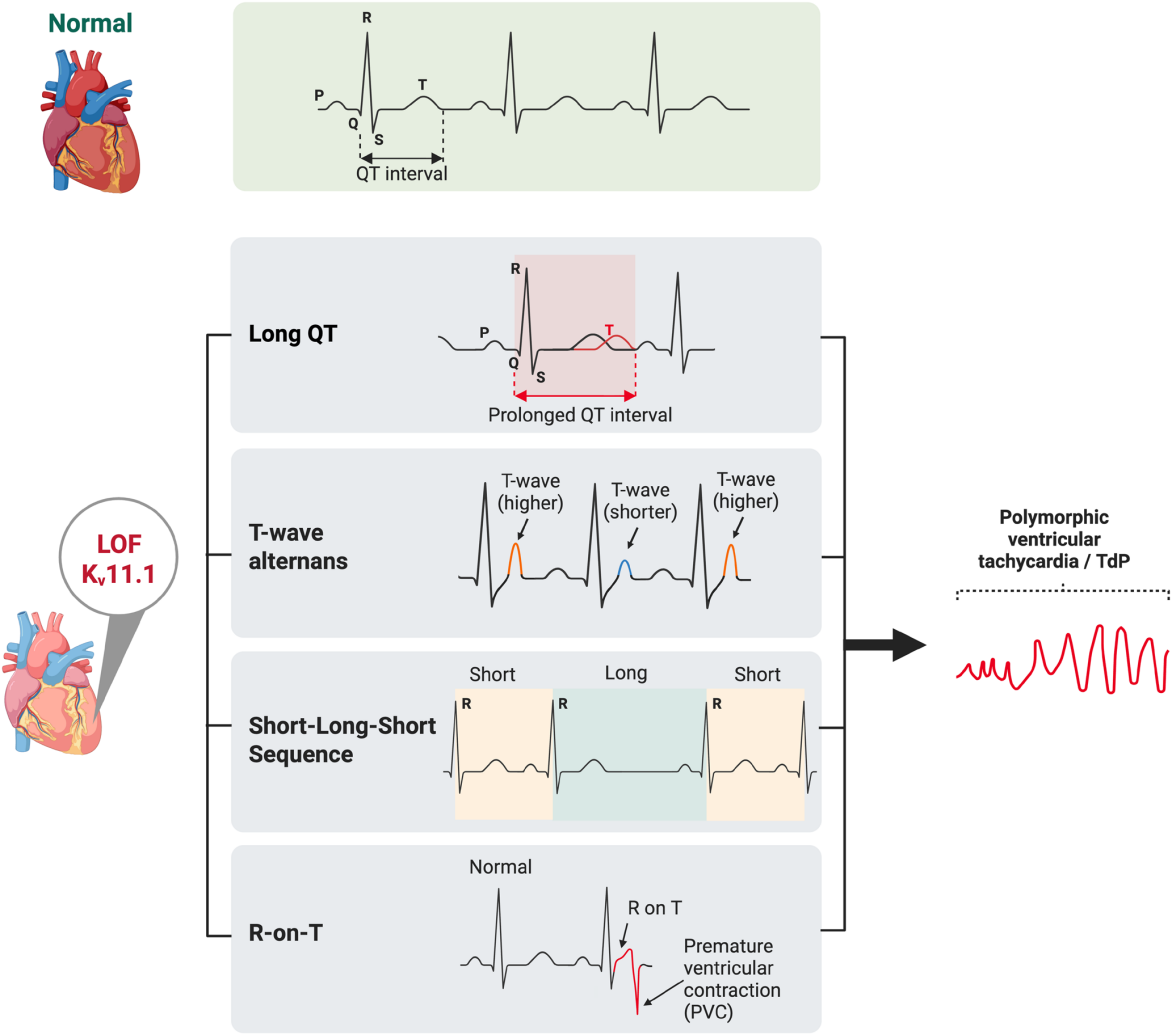
Schematic arrhythmia mechanisms triggered by K_v_11.1 loss‐of‐function. Schematic example of electrocardiographic patterns illustrating normal sinus rhythm in the presence of healthy K_v_11.1 channels (top panel). Loss‐of‐function (LOF) K_v_11.1 prolongs cardiac repolarisation, leading to QT interval prolongation on the ECG. This electrical instability promotes T‐wave alternans, short–long–short sequences, and R‐on‐T premature ventricular contractions. These abnormal patterns can trigger early afterdepolarizations, creating a substrate for polymorphic ventricular tachycardia or Torsades de Pointes (TdP).

The reduction in I_Kr_ can occur through multiple mechanisms that broadly fell into trafficking‐defective and gating‐defective loss‐of‐function pathways. Trafficking defects include reduced transcription or mRNA stability, misfolding during biosynthesis, defective assembly of channel subunits, and impaired transportation to the plasma membrane (Delisle et al. 2004; Sanguinetti and Tristani‐Firouzi 2006). These disruptions may result from altered signal sequences, faulty interactions with chaperone proteins, or abnormal post‐translational modifications such as glycosylation (Delisle et al. 2004). In contrast, gating‐defective variants reach the cell surface but exhibit abnormal activation, inactivation, or deactivation kinetics, altered ion selectivity, or reduced conductance, thereby diminishing effective I_Kr_ despite preserved membrane expression (Delisle et al. 2004; Singh and Auerbach 2024). Although both mechanisms converge on prolongation of QT interval and increased risk of life‐threatening arrhythmias (Figure 2), they have distinct biological and therapeutic implications, particularly with respect to pharmacological modulation.

Importantly, in epilepsy, arrhythmia risk associated with K_v_11.1 loss‐of‐function may be further amplified by acquired I_Kr_ inhibition, as several psychiatric and other medications clinically used for comorbidities act as functional K_v_11.1 blockers and influence cardiac repolarisation (Sanguinetti and Tristani‐Firouzi 2006). In individuals with underlying *KCNH2* dysfunction, such additional reduction in repolarisation reserve may further exacerbate QT prolongation and increase susceptibility to dangerous arrhythmias. Thus, while loss‐of‐function *KCNH2*/K_v_11.1 variants directly impair cardiac repolarisation, cardiac vulnerability in epilepsy reflects an interaction between genetic mechanism, drug exposure, and seizure‐related autonomic modulation. The following section discusses how autonomic regulation of cardiac function, particularly in epilepsy, may interact with K_v_11.1 dysfunction to further increase cardiac vulnerability.

### 5.1 Autonomic Regulation of Cardiac Function and Cardiac Vulnerability in Epilepsy

**FIGURE 3:**
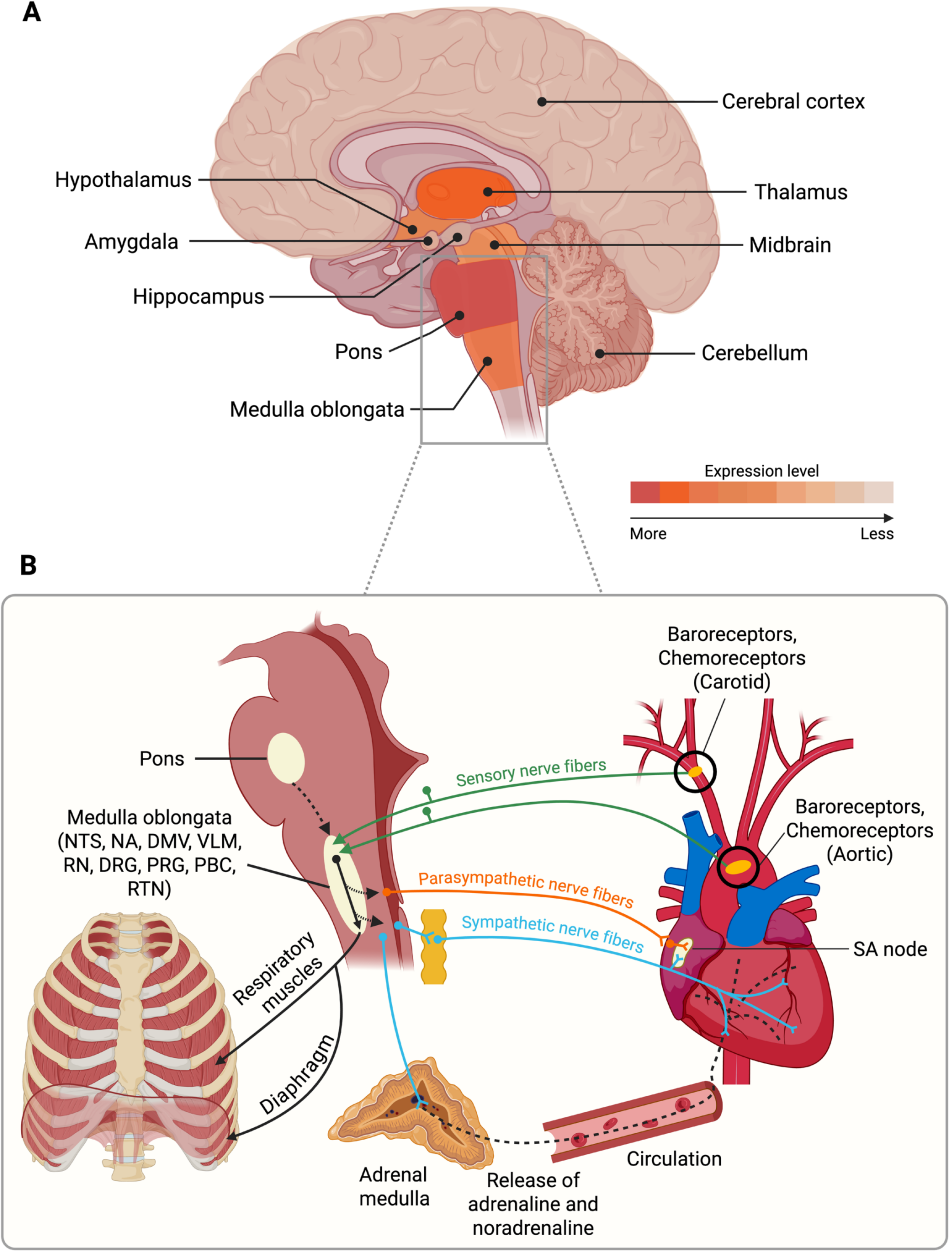
Regional expression of K_v_11.1 channels in the brain and circuits regulating cardiorespiratory function. (A) Heatmap representation of K_v_11.1 expression across major brain regions. Colour gradient indicates expression intensity (dark red = higher, light peach = lower). Heatmap was generated based on expression levels from the Human Protein Atlas (Sjöstedt et al. 2020) (B) Schematic of brainstem and peripheral pathways involved in autonomic and respiratory regulation. The medulla oblongata contains key nuclei including the nucleus tractus solitarius (NTS), nucleus ambiguus (NA), dorsal motor nucleus of the vagus (DMV), ventrolateral medulla (VLM), raphe nuclei (RN), dorsal respiratory group (DRG), parafacial respiratory group (PRG), pre‐Bötzinger complex (PBC), and retrotrapezoid nucleus (RTN). These centres integrate sensory input (green) from carotid and aortic baroreceptors and chemoreceptors and modulate cardiovascular and respiratory function through parasympathetic (orange) and sympathetic nerve fibres (blue). The efferent fibres project to the heart, adrenal medulla, and respiratory muscles, thereby influencing heart rate, contractility, blood pressure, and breathing.

The autonomic nervous system plays a central role in regulating cardiac function through its sympathetic and parasympathetic branches (Figure 3A,B). Sympathetic activation increases heart rate and myocardial excitability via catecholamine release, primarily noradrenaline, while parasympathetic stimulation via the vagus nerve slows heart rate and stabilises repolarisation (Franciosi et al. 2017). Dysregulation of this balance can destabilise cardiac electrophysiology, particularly in individuals with underlying ion channel dysfunction (Franciosi et al. 2017).

**FIGURE 4:**
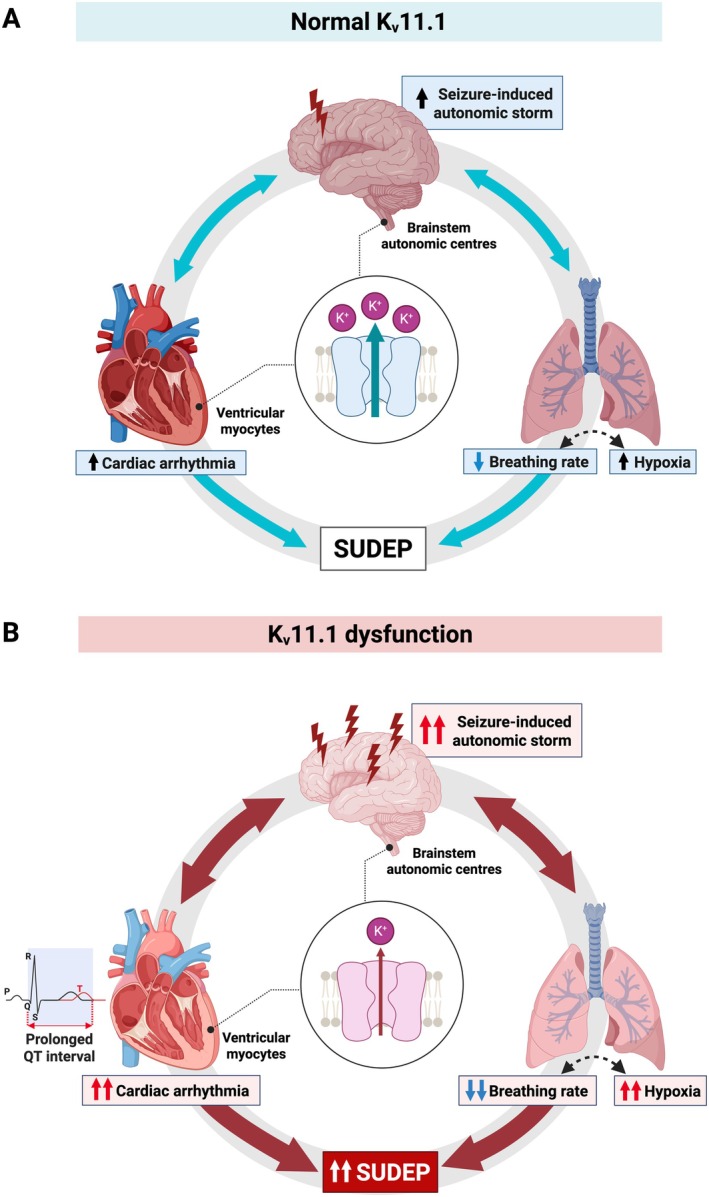
Proposed convergent impact of K_v_11.1 function on SUDEP risk through brain‐heart‐lung interactions. (A) Under normal K_v_11.1 function, a seizure impairs autonomic control in the central nervous system, causing transient changes in cardiac rhythm and breathing. These systems are tightly interdependent, as cardiac arrhythmia can impair oxygen delivery, while respiratory changes influence cardiac excitability. However, compensatory mechanisms, such as enhanced ventilation to minimise hypoxia and autonomic rebalancing to maintain cardiac function, generally stabilises these vital functions and limit SUDEP risk. (B) K_v_11.1 dysfunction amplifies seizure‐induced autonomic disturbances via its expression in the brain, while its expression in the heart further prolongs QT interval and increases arrhythmia susceptibility. Seizure‐induced impaired breathing aggravates systemic hypoxia, which severely destabilises cardiac electrophysiology and triggers life‐threatening arrhythmias that critically compromise cerebral blood flow. This convergence of interdependent dysfunction creates a vicious cycle that markedly elevates SUDEP risk in the presence of loss‐of‐function K_v_11.1.

In LQT2, life‐threatening cardiac events are often triggered by heightened sympathetic drive under certain circumstances, such as auditory stimuli (Schwartz et al. 2001; Chin and Ng 2023). This is particularly relevant in epilepsy, where generalised tonic–clonic seizures can also trigger a ‘sympathetic storm’ via central autonomic pathways, leading to surges in catecholamines (Figures 3A,B and 4) (Devinsky 2004; Nei 2009). These surges enhance L‐type calcium currents and steepen repolarisation gradients in a subset of cardiomyocytes, leading to QT prolongation and an increased susceptibility to cardiac electrical instability (Ochi 1993; Devinsky 2004; Liu et al. 2019). Beyond these electrophysiological effects, symptomatic LQT2 patients exhibit particularly pronounced prolongation of myocardial contraction and marked transmural mechanical dispersion, where the inner heart muscle layers contract longer than the mid‐wall layers (Haugaa et al. 2010). These abnormalities reflect underlying repolarisation defects caused by faulty ion channels including K_v_11.1. This electrical–mechanical mismatch creates a vulnerable period during which afterdepolarisations or after‐contractions can occur and trigger arrhythmias, underscoring that LQT2 disrupts electromechanical coupling. Supporting the clinical evidence, chronic transgenic LQT2 rabbit model demonstrates similar electrical–mechanical abnormalities, as repolarisation was both prolonged and uneven, accompanied by impaired diastolic relaxation (Odening et al. 2013), This parallel between human and animal studies shows that loss of *KCNH2*/K_v_11.1 function directly impairs cardiac mechanics, reinforcing the link between repolarisation defects and mechanical dysfunction.

Given this intrinsically unstable electromechanical substrate in LQT2, seizure‐related sympathetic surges pose an even greater arrhythmic threat. This heightened vulnerability is clinically relevant, as seizure‐induced arrhythmias are a significant cause of mortality in epilepsy. This is supported by a study showing that paediatric patients with both seizures and arrhythmias had substantially higher death rates (Cong et al. 2025). In epilepsy patients with impaired K_v_11.1 function, the combination of impaired cardiac repolarisation and seizure‐induced autonomic neurochemical surges can be particularly dangerous, as the QT interval is further prolonged, increasing the risk of life‐threatening arrhythmias, such as ventricular fibrillation and TdP, and consequently elevating mortality risk (Figure 2) (Singh et al. 2025; Soh et al. 2021; Liu et al. 2019; Schwartz et al. 2012; Smith et al. 2016).

Such LQT2‐ and/or seizure‐mediated arrhythmogenic vulnerability is often preceded by characteristic changes on the ECG (Figure 2). For instance, electrical instability may first appear as changes in T‐wave shape between heartbeats, known as T‐wave alternans, which signifies uneven recovery of the cardiomyocytes after each beat (Figure 2) (Liu et al. 2019). Another warning pattern is the short‐long‐short sequence, where the timing between heartbeats follows a pattern: short pause, then a long one, followed by another short beat (Figure 2) (Liu et al. 2019). A particularly risky ECG abnormality is the R‐from‐T mechanism, which can trigger arrhythmias. This occurs when a premature heartbeat, called a premature ventricular contraction, happens during the vulnerable phase of the heart's recovery, specifically on the downslope of the T‐wave (Figure 2) (Liu et al. 2019; Zhang et al. 2021). During this phase, the heart is not fully reset and is most susceptible to a chaotic rhythm. In hearts with reduced K_v_11.1 function, this combination of ECG abnormalities can lower the threshold for polymorphic ventricular tachycardia or TdP (Liu et al. 2019; Zhang et al. 2021), particularly under conditions of seizure‐mediated autonomic stress or hypoxia (Figure 2). These arrhythmias are well‐established causes of sudden cardiac death, and experimental models have demonstrated that seizures can precipitate such events in the presence of QT prolongation (van der Linde et al. 2021). Although the limited number of SUDEP cases observed in epilepsy monitoring units are predominantly characterised by primary respiratory arrest, accompanying ECG recordings indicate that cardiac rhythm disturbances frequently emerge within the broader cascade of cardiorespiratory failure (Ryvlin et al. 2013; Vilella et al. 2019). These findings suggest that arrhythmias may contribute to SUDEP in genetically or physiologically susceptible individuals, rather than representing a single dominant terminal mechanism.

Given the difficulty of capturing SUDEP events in humans, animal models provide an opportunity to interrogate this susceptibility, under controlled conditions, how seizure‐induced autonomic imbalance interacts with underlying cardiac repolarisation defects. In a rabbit model of *KCNH2‐*LQT2‐SUDEP, seizure episodes were followed by post‐ictal generalised EEG suppression, bradycardia, T‐wave inversion, and focal ventricular activity, leading to asystole and death (Singh et al. 2025). Notably, the corrected QT and JT intervals were significantly prolonged even during interictal periods, and further exacerbated post‐seizure, suggesting that autonomic dysregulation can amplify the arrhythmogenic potential of *KCNH2* variants (Singh et al. 2025). Similarly, double mutant SUDEP mouse models combining epilepsy with loss‐of‐function *Kcnh2* demonstrated impaired cardiac autonomic function, which increased susceptibility to seizure‐induced arrhythmias and mortality risk (Soh et al. 2025). Interestingly, treatment with atenolol, a cardiac‐selective β‐blocker that attenuates sympathetic response, reduced arrhythmia rates and improved survival outcome in these models. These findings from animal models provide compelling evidence that K_v_11.1 impairment due to *KCNH2* variants, when combined with autonomic dysfunction, can increase vulnerability to seizure‐induced arrhythmias and sudden death (Figure 4).

Together, we propose a model in which loss‐of‐function *KCNH2* variants act as a cardiac risk factor for SUDEP, particularly under conditions of autonomic stress such as seizure‐induced sympathetic activation (Figure 4). This interaction between genetically‐impaired repolarisation and seizure‐induced sympathetic surges could be nonlinear and synergistic, meaning that even subclinical *KCNH2* variants may pose significant SUDEP risk when combined with autonomic stressors (Bleakley et al. 2020; Soh et al. 2025). Importantly, this vulnerability may not require structural heart disease or overt epilepsy severity. Instead, it reflects a dynamic vulnerability including K_v_11.1 dysfunction and neurochemical triggers, especially a sympathetic surge, that converge to destabilise cardiac electrophysiology. This mechanistic insight underscores the importance of monitoring autonomic and cardiac function in epilepsy patients, particularly those with known or suspected pathogenic cardiac ion channel variants. It also opens avenues for targeted prophylactic strategies aimed at restoring autonomic balance to mitigate SUDEP risk in genetically susceptible individuals. These include β‐blockade which can attenuate excessive sympathetic activation and stabilise heart function (Soh et al. 2025; Scattolini et al. 2013), and L‐type calcium channel modulation to counteract seizure‐induced catecholamine‐driven calcium influx that exacerbates QT prolongation.

## 6 K_v_11.1 Channel Impairment in the Brain: Another Potential Pathway to SUDEP

The cardiac role of K_v_11.1 channels in arrhythmogenesis and sudden cardiac death is well established, supported by LQT2 and SUDEP‐relevant models demonstrating cardiac electrophysiological abnormalities. In contrast, while emerging evidence suggests that K_v_11.1 dysfunction may also contribute to autonomic and cardiorespiratory dysregulation and SUDEP through neuronal mechanisms (Singh et al. 2025), these central contributions remain largely inferential. K_v_11.1 is expressed throughout the central nervous system where it contributes to the regulation of neuronal excitability and firing dynamics (Sjöstedt et al. 2020; Uhlén et al. 2015; Chiesa et al. 1997). Notably, this includes brain regions that govern autonomic and cardiorespiratory regulation (Table 2, Figure 3A,B). In this section, we first examine how the central nervous system regulates cardiorespiratory homeostasis and how seizures can disrupt this autonomic balance. Building on this, we then explore the potential neuronal contributions of K_v_11.1 dysfunction to SUDEP, focusing on its role in enhancing seizure susceptibility, its expression in brain regions critical for autonomic regulation, and how its impairment may hinder recovery following ictal events.

**TABLE 2:** Expression of *KCNH2*/K_v_11.1 in various brain regions of the central nervous system that regulate cardiorespiratory control.

Region	K_v_11.1 expression level (nTPM)	Function of different brain regions in cardiorespiratory control	References
Brainstem (medulla oblongata)			
Nucleus tractus solitarii	11.4	Integrates baroreceptor/chemoreceptor input; modulates vagal tone and autonomic balance.	Boron and Boulpaep (2016), Betts et al. (2013), Zoccal et al. (2014), Ruffinazzi and Dusi (2021), and Andresen and Paton (2011)
Nucleus ambiguus	11.9	Parasympathetic control of heart rate via vagus nerve.	Petko and Tadi (2025), Ruffinazzi and Dusi (2021), Betts et al. (2013), and Boron and Boulpaep (2016)
Dorsal motor nucleus of vagus	10.0	Regulates parasympathetic output to heart and viscera.	Betts et al. (2013) and Boron and Boulpaep (2016)
Ventrolateral medulla	14.3	Caudal regions maintain autonomic control by inhibiting excessive sympathetic activity.	Sved et al. (2000) and Cravo and Morrison (1993)
		Rostral regions drive sympathetic outflow to regulate blood pressure and heart rate. Also includes dorsal/parafacial respiratory group (supports active expiration and pre‐inspiration); pre‐Bötzinger complex (primary respiratory rhythm generator), and retrotrapezoid nucleus (chemosensory integration and regulates respiratory response to CO_2_).	Oshima et al. (2008), Ruffinazzi and Dusi 2021, Valenza et al. (2025), Alheid and McCrimmon (2008), Forster (2006), Fortuna et al. (2008), Ikeda et al. (2017), Vita et al. (2023), and Hodges and Richerson (2010)
Raphe nuclei	4.9–23.5	Serotonergic regulation of respiratory rhythm and arousal.	Monti (2010), Andersen et al. (2006), Pilowsky (2014), and Ptak et al. (2009)
Brainstem (other regions)			
Pons	23.0	Modulates respiratory phase transitions.	Ikeda et al. (2017), Vita et al. (2023), Alheid and McCrimmon (2008), and Davies and Moores (2010)
Midbrain and diencephalon			
Periaqueductal grey	5.4–9.7	Involved in respiratory recovery and autonomic integration.	Faingold and Feng (2023), Alheid and McCrimmon (2008), and Vita et al. (2023)
Hypothalamus	14.8	Coordinates neuroendocrine responses; regulates cardiovascular and respiratory homeostasis.	Alheid and McCrimmon (2008) and Rahmouni (2016)
Cortical and limbic regions			
Cortex	9.0	Higher‐order modulation of autonomic output.	Betts et al. (2013), Boron and Boulpaep (2016), and Wannamaker (1985)
Amygdala	9.8	Can trigger central apnoea during seizures; modulates autonomic responses.	Nobis et al. (2019) and Dlouhy et al. (2015)
Hippocampus	5.6	Influences autonomic regulation via limbic circuits.	Wandschneider et al. (2015)

*Note:* nTPM (normalised transcript per million), based on the Human Protein Atlas (Sjöstedt et al. 2020). nTPM values are derived from RNA‐seq datasets and may not correspond directly to protein levels.

### 6.1 Central Regulation of Cardiorespiratory Function

The brainstem is a multifunctional structure essential for maintaining vital physiological functions, with anatomically distinct regions contributing to different aspects of autonomic cardiovascular and respiratory control (Table 2, Figure 3A,B). In this section, we outline the roles of key brainstem centres to establish how their coordinated activity supports homeostasis, and how their dysfunction may contribute to SUDEP.

The brainstem integrates sensory input and regulates autonomic output through a network of specialised nuclei (Ruffinazzi and Dusi 2021; Aiba and Noebels 2015; Betts et al. 2013). These include the nucleus tractus solitarii, nucleus ambiguous, dorsal motor nucleus of the vagus, and vasomotor centres in the rostral and caudal ventrolateral medulla (Table 2, Figure 3B). These centres receive afferent input from baroreceptors and chemoreceptors, which are processed by the nucleus tractus solitarii and relayed to downstream nuclei to generate parasympathetic output through the vagus nerve, modulating heart rate and respiratory rhythm (Figure 3B) (Boron and Boulpaep 2016; Betts et al. 2013; Petko and Tadi 2025; Zoccal et al. 2014; Tolstykh and Cavazos 2013; Ruffinazzi and Dusi 2021; Andresen and Paton 2011). Specifically, acetylcholine released at the cardiac sinoatrial ganglia reduces pacemaker activity and slows the heart rate (Akunna and Abah 2023; Ruffinazzi and Dusi 2021). In contrast, the rostral ventrolateral medulla activates sympathetic preganglionic neurons in the spinal cord (Oshima et al. 2008). These neurons then trigger the release of noradrenaline in the heart, which activates β1‐adrenergic receptors to increase heart rate and contractility. Simultaneously, sympathetic stimulation from the preganglionic neurons causes blood vessel constriction by activating α‐adrenergic receptors, helping to raise or maintain blood pressure (Ruffinazzi and Dusi 2021; Valenza et al. 2025). Collectively, these centres modulate parasympathetic and sympathetic outputs essential for cardiovascular and respiratory stability.

In addition to direct autonomic control, brainstem regions such as the caudal ventrolateral medulla and nucleus tractus solitarii contribute to broader regulatory functions (Table 2). The caudal ventrolateral medulla helps to maintain autonomic balance by inhibiting excessive sympathetic activity driven by the rostral ventrolateral medulla, primarily through GABAergic neurotransmission (Sved et al. 2000; Cravo and Morrison 1993). Meanwhile, the nucleus tractus solitarii communicates with cortical, subcortical, and hypothalamic regions, enabling both rapid autonomic responses and slower neuroendocrine adjustments (Forstenpointner et al. 2022; Ruffinazzi and Dusi 2021). Through its connection with the paraventricular nucleus of the hypothalamus, the nucleus tractus solitarii activates the hypothalamic–pituitary–adrenal axis, releasing hormones such as cortisol, adrenaline, and vasopressin (Murphy et al. 2023). These hormones help to stabilise cardiovascular, respiratory, and metabolic functions by supporting vascular tone and fluid balance in response to physiological stress (Rivier and Vale 1983; De Winter et al. 2003). This integration between autonomic and neuroendocrine systems is therefore important for ensuring a coordinated physiological recovery, especially following seizure activity.

The brainstem also houses specialised centres responsible for generating and modulating respiratory rhythms (Table 2, Figure 3B). The pre‐Bötzinger complex, located in the ventrolateral medulla, is widely recognised as the primary respiratory rhythm generator in mammals (Forster 2006; Ikeda et al. 2017; Vita et al. 2023; Alheid and McCrimmon 2008). It modulates a variety of input such as glutamatergic, glycinergic, serotonergic and cholinergic networks, and expresses neurochemicals such as somatostatin and neurokinin‐1 receptors, as well as adrenergic C1 chemoreceptors to generate and maintain autonomous rhythmic breathing (Carrick et al. 2025; Vita et al. 2023; Patodia et al. 2018). This complex works in concert with other respiratory centres, including (1) the parafacial respiratory group, which supports pre‐inspiratory and active expiratory phases, especially under conditions of increased respiratory demand such as exercise (Fortuna et al. 2008; Ikeda et al. 2017; Korsak et al. 2018; Vita et al. 2023; Alheid and McCrimmon 2008); (2) the pons, which modulates respiratory patterns and transitions between phases (Ikeda et al. 2017; Vita et al. 2023; Alheid and McCrimmon 2008; Davies and Moores 2010); (3) the dorsal respiratory group within the nucleus tractus solitarii, which regulates spontaneous inspiratory rhythm (Alheid and McCrimmon 2008, Davies and Moores 2010); and (4) the raphe nuclei, distributed throughout the brainstem, which contain serotonergic and substance‐P‐binding neurokinin‐1‐expressing neurons that promote wakefulness and stabilise respiratory rhythm (Monti 2010; Andersen et al. 2006; Pilowsky 2014; Ptak et al. 2009). These centres collectively maintain rhythmic breathing and adapt respiratory patterns to meet changing physiological demands.

Together, these interconnected brain centres coordinate a complex network of neurotransmitters and neuropeptides to regulate autonomic output and maintain homeostasis (Figure 3). Disruption of these systems, whether by seizures and/or underlying channelopathies, can severely compromise the brain's ability to preserve cardiorespiratory stability, potentially contributing to SUDEP risk. As summarised in Table 2, *KCNH2*/K_v_11.1 is expressed in key centres that coordinate cardiorespiratory homeostasis, including the brainstem medullary (nucleus tractus solitarii, nucleus ambiguus, dorsal motor nucleus of vagus, ventrolateral medulla, raphe nuclei) and pontine centres, as well as midbrain (periaqueductal grey), diencephalic (hypothalamus), and cortical–limbic regions (cortex, amygdala, hippocampus). This distribution therefore places K_v_11.1 within interconnected circuits that (i) enhance vagal tone and restrain sympathetic drive during recovery (nucleus tractus solitarii, nucleus ambiguous, caudal ventrolateral medulla, dorsal motor nucleus of vagus), (ii) regulate sympathetic outflow to the heart and vasculature (rostral ventrolateral medulla, hypothalamus), and (iii) generate and modulate respiratory rhythm (pre‐Bötzinger complex, parafacial group, pons, raphe nuclei, periaqueductal grey; Figure 3B). Higher‐order cortical and limbic regions, including amygdala and hippocampus, provide modulatory control over these brainstem networks. Consequently, disruptions of K_v_11.1 function in these regions could influence whether seizure‐related autonomic disturbances progress toward recovery or deteriorate into cardiorespiratory collapse. However, studies directly examining how K_v_11.1 dysfunction affects these regions remain limited. In the following sections, we review how seizures impair cardiorespiratory control within these circuits and consider how *KCNH2*/Kv11.1 expression may exacerbate seizure‐induced dysfunction and prolong recovery.

### 6.2 Seizure‐Induced Disruption of Autonomic Regulation

Seizures can significantly impair the brain's ability to regulate cardiovascular and respiratory homeostasis by interfering with the normal function of brainstem autonomic centres. During seizures, abnormal cortical discharges can propagate to the nucleus tractus solitarii, nucleus ambiguus, dorsal motor nucleus of the vagus, and ventrolateral medulla, leading to acute and chronic abnormalities (Wannamaker 1985; Heath 1976; Tolstykh and Cavazos 2013; Zoccal et al. 2014; Aiba and Noebels 2015). One acute mechanism is seizure‐induced spreading depolarisation, a wave of sustained neuronal silencing across brainstem centres, which might act as a protective mechanism to limit seizure spread but can also trigger cardiorespiratory arrest (Hübel et al. 2017; Hinzman et al. 2016; Mulkey and Milla 2022; Aiba and Noebels 2015). Over time, repeated seizures can cause progressive neuronal loss in these regions, weakening their ability to maintain cardiovascular and respiratory homeostasis (Wannamaker 1985; Heath 1976; Tolstykh and Cavazos 2013; Zoccal et al. 2014; Aiba and Noebels 2015). This includes impairment in the nucleus tractus solitarii's role in enhancing vagal tone and suppressing sympathetic hyperactivity during and after seizures (Table 2) (Andresen and Paton 2011; Forstenpointner et al. 2022; Lamy 2012; Martinez and Kline 2021; Zoccal et al. 2014). When this recovery mechanism is compromised, it can prolong autonomic instability and facilitate a cascade of events such as central apnoea, bradycardia and post‐ictal EEG suppression, all of which are associated with increased SUDEP risk (Ryvlin et al. 2013; Devinsky et al. 2016).

Seizures can also disrupt the delicate balance between sympathetic and parasympathetic output. Under normal conditions, the caudal ventrolateral medulla suppresses excessive sympathetic output from the rostral ventrolateral medulla via GABAergic signalling (Table 2) (Sved et al. 2000; Cravo and Morrison 1993). However, during sympathetic stress such as seizures, this inhibitory control can fail. The resulting disinhibition allows bulbospinal neurons to drive excessive sympathetic outflow to the heart and blood vessels (Valenza et al. 2025; Cheng et al. 2019). This cascade of events results in elevated noradrenaline release, increased L‐type calcium currents and stronger myocardial contractility (Ochi 1993; Devinsky 2004; Liu et al. 2019). This excessive calcium influx steepens repolarisation gradients, destabilising cardiac rhythm and triggering life‐threatening arrhythmias and cardiac arrest.

Respiratory centres, including the pre‐Bötzinger complex are similarly vulnerable to seizure‐induced dysfunction (Table 2). Post‐mortem studies in SUDEP and sudden infant death cases have revealed neuronal alterations in this region, especially those involving neurokinin‐1 and somatostatin receptors (Patodia et al. 2018; Lavezzi and Matturri 2008). Dysfunction in the pre‐Bötzinger complex, whether due to direct seizure impact, impaired serotonergic input, or ion channel abnormalities (e.g., *SCN8A*, *KCNQ*) that impair neuronal excitability supporting respiratory rhythm generation, can lead to central apnoea and fatal respiratory failure (Mir et al. 2025). This is further supported by experimental models, which demonstrated that serotonergic modulation of the pre‐Bötzinger complex can reduce seizure‐induced respiratory arrest, highlighting its role in postictal respiratory recovery (Ma et al. 2022; Ma et al. 2020; Vilella et al. 2019). In the amygdala, seizures can also trigger central apnoea by inhibiting crucial brainstem respiratory control centres (Nobis et al. 2019; Dlouhy et al. 2015). Other respiratory centres, including the parafacial respiratory group, pons, dorsal respiratory group, and raphe nuclei, also contribute to this vulnerability during seizures. These regions coordinate respiratory phase transitions, inspiratory rhythm, and chemosensitivity to CO_2_ (Table 2) (Ikeda et al. 2017; Vita et al. 2023; Hodges and Richerson 2010; Richerson 2004). Recent clinical evidence highlights seizure‐induced apnoea as a critical mechanism contributing to SUDEP, reinforcing the role of brainstem respiratory centres in ictal and postictal vulnerability (Ryvlin 2025; Magana‐Tellez et al. 2025; Ochoa‐Urrea et al. 2025).

In summary, seizures disrupt the brain's autonomic centres, silencing neurons and disrupting key circuits that regulate cardiovascular function and breathing. This impairs parasympathetic recovery and sympathetic inhibition, thereby increasing the risk of central apnoea and arrhythmias. Although evidence is lacking, given that *KCNH2*/K_v_11.1 is expressed in the autonomic and cardiorespiratory control regions, including brainstem sympathetic and parasympathetic nuclei, ventrolateral medulla, pons, midbrain, diencephalon, and higher‐order cortico‐limbic modulatory areas (Table 2), we propose that K_v_11.1 dysfunction within these interconnected networks may amplify seizure‐induced vulnerabilities (Figures 3 and 4). In particular, reduced K_v_11.1 function may exacerbate seizure‐related neuronal dysfunction and further impair parasympathetic recovery, leading to excessive sympathetic output and increasing susceptibility to postictal respiratory failure and malignant arrhythmias. Further studies are needed to directly test this hypothesis. In the next section, we discuss how K_v_11.1‐mediated regulation of neuronal firing properties may act as a modifier of seizure threshold, influence seizure‐related vulnerabilities, and in turn potentially exacerbate mortality risk.

### 6.3 K_v_11.1 Increasing Seizure Susceptibility: A Potential Risk Modifier

Changes in K_v_11.1 function due to loss of *KCNH2* function can increase neuronal excitability and lower seizure thresholds. The channel is expressed in key regions of the brain including the cortex, hippocampus and brainstem (Table 2, Figure 3A) (Sjöstedt et al. 2020; Uhlén et al. 2015), where it contributes to I_Kr_. K_v_11.1's unique biophysical properties—slow activation and rapid inactivation—help stabilise neuronal firing by hyperpolarising the resting membrane potential and limiting repetitive action potential generation (Chiesa et al. 1997). Genetic evidence from conditional *Kcnh2* knockout models indicates that K_v_11.1 contributes primarily to suprathreshold neuronal functions, with loss of I_Kr_ altering firing behaviour during ongoing activity (Schwarz et al. 2024). Complementing this, pharmacological studies across multiple neuronal types show that inhibiting K_v_11.1 depolarises the resting membrane potential and increases both intrinsic excitability and spontaneous firing (Hirdes et al. 2009; Ji et al. 2012; Sacco et al. 2003). These findings suggest that reduced I_Kr_ from loss of K_v_11.1 function could both facilitate burst escalation and lower the threshold for action‐potential initiation, thereby increasing seizure susceptibility. As I_Kr_ channels deactivate slowly, an increase in their conductance can offset sodium ion entry, which then prolongs the time between successive neuronal spikes (Chiesa et al. 1997; Singh and Auerbach 2024). As such, inhibition of I_Kr_ shortens these inter‐spike intervals, diminishing spike‐frequency adaptation and facilitating burst‐like neuronal firing. All these lead to the neurons becoming hyperexcitable, therefore lowering the seizure threshold, increasing action potential firing frequency, and facilitating seizure propagation (Papa et al. 2003; Singh and Auerbach 2024).

In addition to its neuronal role, K_v_11.1 channels are also expressed in glial cells, including astrocytes and microglia, which are increasingly recognised for their role in seizure modulation (Emmi et al. 2000; Linden 1997). Although glial cells themselves do not fire action potentials, they regulate extracellular ion concentrations, neurotransmitter clearance and inflammatory responses, all of which could influence neuronal excitability (Bergles and Jahr 1998; Syková and Chvátal 1993; Kim et al. 2016). As such, K_v_11.1 dysfunction in glial cells could impair ionic buffering and glutamate uptake, resulting in sustained neuronal depolarisation and increased excitability. Furthermore, loss of glial K_v_11.1 function could exacerbate neuroinflammation, a known contributor to seizure generation (Hollis and Lukens 2025; Sanz and Garcia‐Gimeno 2020). These non‐neuronal mechanisms suggest that glial K_v_11.1 may also act as an indirect but important modifier of seizure susceptibility.

To further add to complexity, a primate‐specific, brain‐selective isoform of *KCNH2*, known as *KCNH2*‐3.1, has also been identified in the cortex and hippocampus, regions central to seizure generation. This isoform lacks the PAS domain found in canonical K_v_11.1 channels, resulting in altered channel kinetics that promote faster deactivation and increased neuronal firing rates (Sanchez‐Conde et al. 2022). These changes in excitability have been linked to increased seizure susceptibility, cognitive dysfunction, and psychiatric conditions, particularly schizophrenia (Huffaker et al. 2009).

In rodent models, pharmacological blockade of I_Kr_ has been shown to increase spontaneous firing and reduce spike‐frequency adaptation in various neuronal populations, including midbrain dopaminergic neurons, layer V cortical pyramidal neurons, and excitatory and inhibitory neurons in the vestibular nuclei (Sanchez‐Conde et al. 2022; Ji et al. 2012; Pessia et al. 2008). These findings indicate that K_v_11.1‐mediated currents play an important role in stabilising neuronal firing during prolonged activation. Such firing‐stabilising properties are likely critical in rhythm‐generating and autonomic circuits for maintaining coherent output under conditions of heightened synaptic drive. Accordingly, K_v_11.1 dysfunction in brainstem regions including the nucleus tractus solitarii, ventrolateral medulla, raphe nuclei, and pre‐Bötzinger complex (Table 2) may impair the ability of these circuits to sustain stable cardiorespiratory rhythms during and after seizures, particularly under hypoxic or hypercapnic stress, thereby compromising post‐ictal recovery and increasing vulnerability to fatal outcomes. Consistent with this framework, a recent study using a knock‐in rabbit model with reduced K_v_11.1 function exhibited spontaneous epileptiform activity, seizures, and a significantly higher rate of sudden deaths (Singh et al. 2025). This supports a potential link between K_v_11.1 dysfunction, seizure susceptibility and SUDEP. However, contrasting evidence from epilepsy mouse models showed that loss of K_v_11.1 function did not significantly alter spontaneous seizure frequency or baseline neuronal excitability, despite a significant increase in seizure‐related mortality (Soh et al. 2025). The lack of impact on seizure characteristics suggests that K_v_11.1 dysfunction alone is unlikely to be a direct cause of epilepsy. However, increased seizure‐related mortality in the mouse models, along with hyperexcitability observed in other animal models, indicates that K_v_11.1 may act more as a vulnerability factor that compromises network stability and recovery during seizures. Further preclinical studies are needed to directly test how K_v_11.1 dysfunction within autonomic circuits contributes to seizure‐mediated mortality and SUDEP risk.

Clinically, *KCNH2* variants are linked to a significantly increased risk of seizure‐like episodes, particularly in LQT2 patients. For example, 39% of LQT2 patients have been reported to have a history of seizures or epilepsy, which is significantly higher compared to other LQTS subtypes (10%) (Johnson et al. 2009). Another study corroborated this, with epilepsy diagnosed in 7 out of 190 (3.7%) LQT2 patients, compared to 0.7% in LQT1 and 2.0% in LQT3 (Anderson et al. 2014). Seizure‐like episodes were also more common in these LQT2 patients (18%) than in other subtypes (8%). Similarly, seizure‐like episodes and EEG abnormalities were also frequently observed in LQT2 patients, although without a diagnosis of epilepsy (González et al. 2018). Taken together, while these findings suggest a strong association between K_v_11.1 dysfunction and seizure susceptibility, genetic evidence linking *KCNH2* to epilepsy remains limited to case reports and small cohort studies (Partemi et al. 2013; Zhou et al. 2023; Zamorano‐León et al. 2012). Moreover, patients with K_v_11.1 dysfunction can experience syncope accompanied by seizure‐like motor activity, which can closely mimic epileptic seizures (Kang et al. 2021; Shin et al. 2019). Convulsive syncope generally results from transient cerebral hypoperfusion secondary to cardiac arrhythmias, and that clinical differentiation from epileptic seizures is often difficult without simultaneous EEG‐ECG recordings (Medford et al. 2014; Galtrey et al. 2019). This overlap often leads to misdiagnosis, as the underlying cause may be cardiac‐mediated rather than true epileptic activity, therefore requiring a different treatment strategy (Medford et al. 2014; Galtrey et al. 2019).

In summary, K_v_11.1 dysfunction can increase neuronal excitability and has been associated with seizure‐like episodes and epilepsy, although evidence from clinical and preclinical models has been inconsistent. This suggests that K_v_11.1 may not be sufficient on its own to drive epileptogenesis but its contribution to seizure susceptibility may be context‐dependent, varying across species, genetic backgrounds, and environmental factors. As increased seizure frequency and severity is a well‐established risk factor for SUDEP, further investigation is warranted to understand how K_v_11.1 dysfunction can influence seizure burden and mortality through its effects on neuronal excitability.

### 6.4 K_v_11.1 Dysfunction Exacerbating Seizure‐Induced Cardiorespiratory Failure Through Multiple Mechanisms

In addition to K_v_11.1's role in regulating neuronal excitability, the channel is also expressed in key autonomic regulatory regions of the brain (Table 2, Figure 3A) (Sjöstedt et al. 2020; Uhlén et al. 2015; Betts et al. 2013). Although reports on K_v_11.1 expression in the periaqueductal grey are contradicting, dysfunction in nearby K_v_11.1‐expressing connected circuits may still affect its function (Faingold and Feng 2023; Sjöstedt et al. 2020). These regions, as discussed above, are essential for maintaining cardiovascular and respiratory homeostasis (Table 2). While a direct causal link between K_v_11.1 dysfunction in these autonomic control regions and SUDEP has yet to be established, its expression in these areas suggests that its dysfunction could have consequences beyond just the heart. Supporting this, studies on other K_v_ channel subtypes in corticolimbic and brainstem areas suggest that K_v_11.1 impairment may similarly disrupt modulatory and integrative circuits involved in cardiorespiratory control (Hu et al. 2025; Paulhus and Glasscock 2024; Trosclair et al. 2020; Dhaibar et al. 2019; Revill et al. 2021). Given that seizures affect brain centres that maintain cardiorespiratory function, K_v_11.1 impairment could further compromise neuronal activity and hinder postictal autonomic recovery. This dual impact on both central autonomic circuits and cardiac electrophysiology represents a converging risk pathway that may significantly elevate SUDEP risk (Figure 4). We propose the following mechanisms through which this convergence may occur.

#### 6.4.1 Compounding Effects of Seizure‐Induced Structural Damage and K_v_11.1 Impairment on SUDEP Risk

One potential pathway for increased SUDEP risk involves the combined impact of seizure‐induced structural brain changes and K_v_11.1 dysfunction within autonomic control regions. Neuroimaging studies provide indirect support of this hypothesis by revealing structural abnormalities in the brains of individuals with epilepsy who later died from SUDEP, particularly in regions where K_v_11.1 is expressed. For instance, one reported more pronounced volume reduction in brainstem regions critical for cardiorespiratory control, including periaqueductal grey, in SUDEP patients compared to those with temporal lobe epilepsy who did not experience SUDEP (Mueller et al. 2014). A subsequent study found that patients with more extensive brainstem atrophy, especially in the raphe nuclei and medulla oblongata, tended to experience SUDEP earlier, suggesting a correlation between structural degeneration and mortality risk (Mueller et al. 2018). Additional evidence observed increased right amygdalo‐hippocampal volume and reduced left thalamic volume in SUDEP cases compared to healthy controls, indicating possible asymmetrical disruptions in autonomic processing (Wandschneider et al. 2015). Thinning of the frontal cortex, a region involved in higher modulation of autonomic output, was also identified in patients at high risk of SUDEP (Allen, Harper, Lhatoo, et al. 2019). Functional and network‐level disruptions, especially in the cortical or subcortical regulatory regions, medulla, pons, and periaqueductal grey, have also been associated with increased SUDEP risk (Mueller et al. 2018; Allen, Harper, Guye, et al. 2019). These changes in the brain are strongly associated with frequent, severe seizures, a well‐known risk factor for SUDEP. Collectively, we hypothesise that K_v_11.1 impairment in these regions, when combined with seizure‐induced structural and functional vulnerabilities such as brainstem atrophy and disrupted autonomic networks, could further compromise postictal autonomic recovery, thus amplifying the risk of SUDEP.

#### 6.4.2 K_v_11.1 Dysfunction Exacerbates Postictal Respiratory and Cardiovascular Vulnerability

Generalised tonic–clonic seizures, as discussed, can compromise central autonomic control, triggering apnoea, hypoventilation, and cardiac arrhythmias (Figure 4) (Blum 2009; Mir et al. 2025). As such, K_v_11.1 abnormality in brain regions critical for cardiorespiratory control could further reduce the system's ability to detect and respond to ictal respiratory and cardiovascular challenges (Aiba and Noebels 2015; Ruffinazzi and Dusi 2021).

In the raphe nuclei, K_v_11.1 dysfunction could impair the release of neuropeptides and reduce sensitivity to CO_2_ and O_2_ (Tao et al. 2010; Buchanan 2019; Auzmendi and Lazarowski 2023; Patodia et al. 2018; Buchanan and Richerson 2010). Additionally, disrupted K_v_11.1 activity may interfere with chemosensory and cardiorespiratory integration in regions like the retrotrapezoid nucleus and nucleus tractus solitarii (Bodineau et al. 2000; Guyenet and Mulkey 2010; Mir et al. 2025). In the amygdala, enhanced neuronal excitability may increase the likelihood of seizure‐induced apnoea (Nobis et al. 2019; Dlouhy et al. 2015), while altered I_Kr_ signaling in the hypothalamus may impair release of hormones that maintain postictal cardiorespiratory function (Alheid and McCrimmon 2008; Rahmouni 2016). The channel's dysfunction could also affect the cortex, periaqueductal grey and other brainstem areas, further limiting the brain's ability to restore stable breathing and cardiovascular function postictally (Fortuna et al. 2008; Ikeda et al. 2017; Korsak et al. 2018; Vita et al. 2023; Alheid and McCrimmon 2008; Davies and Moores 2010; Mir et al. 2025; Aiba and Noebels 2015).

K_v_11.1 dysfunction in the brain could also exacerbate neurochemical imbalances, further delaying postictal cardiorespiratory recovery (Figures 3B and Figure 4). By altering neuronal excitability, reduced I_Kr_ signalling could interfere with serotonergic activation, which is critical for enhancing respiratory drive in response to seizure‐induced hypoxia (Faingold and Feng 2023; Murugesan et al. 2018). Additionally, K_v_11.1 dysfunction could amplify adenosinergic suppression of brainstem respiratory centres, further delaying recovery following seizures (Shen et al. 2010; Faingold and Feng 2023). It may also worsen deficits in GABAergic inhibition, which is already impaired by recurrent seizures (Petroff et al. 1996; Sperk et al. 2004), weakening control over excitatory circuits and contributing to autonomic instability (Sears and Hewett 2021; Sutor and Luhmann 1995).

Taken together, K_v_11.1 impairment across these regions could disrupt neurochemical signalling, dampen chemosensory responsiveness, and exacerbate seizure‐induced cardiorespiratory dysfunction, ultimately compromising postictal recovery and increasing SUDEP risk.

#### 6.4.3 The Interdependence of Neuro‐Cardiovascular‐Respiratory Systems

Emerging evidence supports a mechanism underlying SUDEP caused by a convergence of dysfunction across neuronal, cardiac and respiratory systems (Bagnall et al. 2017; Mir et al. 2025; Singh et al. 2023). These systems are closely interdependent, and disruption in one can rapidly destabilise another, creating a dangerous feedback loop (Figure 4).

During seizures, elevated extracellular potassium and glutamate levels can trigger a wave of electrical disturbance called spreading depolarisation. As mentioned previously, this event briefly silences neurons across brain regions, including those that control cardiorespiratory function, and is seen on EEG as generalised suppression (Hübel et al. 2017; Hinzman et al. 2016; Mulkey and Milla 2022). This silencing of autonomic centres compromises the brain's ability to restore cardiorespiratory homeostasis postictally (Aiba and Noebels 2015). On top of that, seizure‐induced central apnoea leads to hypoxia, which further destabilises cardiac electrophysiology (Mir et al. 2025). When autonomic regulation is suppressed, K_v_11.1 dysfunction within autonomic centres and cardiac tissues can further impair the recovery of respiratory and cardiovascular function. This can lead to prolonged respiratory depression, bradycardia and even asystole, all of which are hallmarks of SUDEP (Aiba and Noebels 2015). Supporting this, continuous video‐EEG monitoring in epilepsy patients who later died of SUDEP commonly revealed ictal and postictal hypoventilation (Bateman et al. 2010; Ryvlin et al. 2013). This respiratory depression led to hypoxemia and acidosis, which in turn impaired cortical recovery and ultimately contributed to cardiac failure (Bateman et al. 2010). These findings reinforce the concept that SUDEP arises from a breakdown in the coordination between central, respiratory, and cardiovascular systems, which may be exacerbated by K_v_11.1 dysfunction (Figure 4).

### 6.5 Summary: A Possible Mechanistic Link to SUDEP Involving K_v_11.1 Dysfunction

All in all, K_v_11.1 channel expression in autonomic control centres and cardiac tissues, along with structural and functional brain abnormalities observed in SUDEP cases and the channel's role in modulating neuronal excitability, supports a pathway to SUDEP: K_v_11.1 dysfunction could reduce seizure thresholds and disrupt coordination between higher cortical regions and brainstem centres during and after seizures, leading to impaired autonomic‐mediated recovery processes, such as respiratory and cardiac stabilisation. Disruptions in each of these systems may trigger a dangerous feedback loop, whereby impaired recovery further destabilises autonomic and cardiorespiratory function, increasing the likelihood of fatal outcomes (Figure 4). Investigating the central role of K_v_11.1 alongside its cardiac effects will be critical to uncovering the mechanisms underlying SUDEP and guiding development of targeted interventions.

## 7 Clinical Implications and Future Directions

Given the multi‐faceted nature of SUDEP, future research should adopt a multi‐systems‐level approach that integrates the complex interactions between neural, respiratory, and cardiac networks (Mbizvo et al. 2025). This framework has implications for risk stratification, genetic evaluation, and management in selected epilepsy populations. While routine genetic screening for all individuals with epilepsy is currently not indicated, consideration of *KCNH2* testing may be reasonable in specific contexts, including unexplained syncope, documented ictal‐related arrhythmias, QT‐interval abnormalities on the ECG, or a personal or family history suggestive of inherited arrhythmia syndromes. In such cases, genetic findings should be interpreted alongside clinical phenotype, ECG features, medication exposure, and family history. Interpretation of *KCNH2* variants of uncertain significance (VUS) remains a challenge and should incorporate longitudinal ECG data, segregation analysis, or functional studies where available.

Beyond genetic testing, development of predictive biomarkers for risk stratification and guided intervention remains a priority, including physiological measures such as QT‐interval or heart rate variability, imaging‐based markers of brainstem structure or connectivity, and molecular indicators such as adenosine or serotonin. Longitudinal studies that combine EEG, ECG, and respiratory monitoring in high‐risk epilepsy patients may further refine risk assessment. Management strategies in individuals with overlapping epilepsy and cardiac risk should be individualised. In ambiguous cases, β‐blockers may be considered to mitigate seizure‐related sympathetic surges, whereas device‐based therapies such as implantable pacemaker‐defibrillators are generally reserved for patients with documented malignant arrhythmias or established inherited arrhythmia syndromes. Additionally, interventions that support autonomic recovery and respiratory stability, including vagal nerve stimulation, respiratory pacing, or targeted neuromodulation, may complement cardiac‐focused strategies. Notably, vagal nerve stimulation has been demonstrated to reduce SUDEP risk by almost 60% (Sveinsson et al. 2020). As K_v_11.1 channel is expressed in both cardiac tissue and central nervous system regions involved in cardiorespiratory control, targeted modulation of this channel may offer dual‐system benefits. Importantly, the insights gained from SUDEP research may also inform our understanding of other sudden death conditions that involve *KCNH2*, such as sudden infant death syndrome (Tfelt‐Hansen et al. 2011; Sweeting and Semsarian 2014), potentially leading to broader applications in sudden death prevention.

## 8 Conclusion

Variation in *KCNH2* may create convergent vulnerability increasing SUDEP risk, where impaired K_v_11.1 channels in both the heart and brain compromise the body's ability to withstand and recover from seizures. This multi‐system failure is not merely additive; it is synergistic, reflecting a breakdown in the coordination between central and peripheral autonomic circuits. By integrating cardiac, neural, and respiratory insights, and focusing on the shared molecular mechanisms such as compromised K_v_11.1 channel activity, there is the potential to improve diagnostics, personalise therapies and reduce SUDEP incidence. This framework also broadens our understanding of other sudden death syndromes involving variation in *KCNH2*, paving the way for cross‐disciplinary collaboration to advance both epilepsy care and our understanding of multisystem vulnerabilities.

## Author Contributions

**Hian M. Lee:** visualization, writing – original draft. **Xue N. Gan:** visualization, writing – review and editing. **Khaing Phyu Aung:** visualization, writing – review and editing. **Ian C. Forster:** writing – review and editing. **Christopher A. Reid:** conceptualization, supervision, visualization, writing – review and editing, writing – original draft, resources. **Ming S. Soh:** conceptualization, writing – original draft, visualization, writing – review and editing, supervision, resources.

## Funding

This work was supported by Anonymous philanthropic gift. Medical Research Future Fund, 2016012. Citizens United for Research in Epilepsy. National Health and Medical Research Council, 2019804.

## Conflicts of Interest

The authors declare no conflicts of interest.

## Data Availability

The authors have nothing to report.
